# Chloroplast thioredoxin systems dynamically regulate photosynthesis in plants

**DOI:** 10.1042/BCJ20180707

**Published:** 2019-04-15

**Authors:** Lauri Nikkanen, Eevi Rintamäki

**Affiliations:** Molecular Plant Biology, Department of Biochemistry, University of Turku, FI-20014 Turku, Finland

**Keywords:** Calvin cycle, fluctuating light, metabolic regulation, NTRC, redox homeostasis, thylakoid electron flow

## Abstract

Photosynthesis is a highly regulated process in photoautotrophic cells. The main goal of the regulation is to keep the basic photosynthetic reactions, i.e. capturing light energy, conversion into chemical energy and production of carbohydrates, in balance. The rationale behind the evolution of strong regulation mechanisms is to keep photosynthesis functional under all conditions encountered by sessile plants during their lifetimes. The regulatory mechanisms may, however, also impair photosynthetic efficiency by overriding the photosynthetic reactions in controlled environments like crop fields or bioreactors, where light energy could be used for production of sugars instead of dissipation as heat and down-regulation of carbon fixation. The plant chloroplast has a high number of regulatory proteins called thioredoxins (TRX), which control the function of chloroplasts from biogenesis and assembly of chloroplast machinery to light and carbon fixation reactions as well as photoprotective mechanisms. Here, we review the current knowledge of regulation of photosynthesis by chloroplast TRXs and assess the prospect of improving plant photosynthetic efficiency by modification of chloroplast thioredoxin systems.

## Introduction

In nature, light intensity is constantly changing in plant growth habitats, including both seasonal alteration of daily light period and daily fluctuation of light intensity due to cloudiness and other environmental factors. Optimization of photosynthetic production under fluctuating light conditions needs balancing of photosynthetic reactions by induction of regulatory mechanisms. The photosynthetic reactions also produce highly energetic intermediates that are dangerous to cellular infrastructure and function, if they are not consumed/processed correctly by photoprotective mechanisms. The mechanisms to balance light capture and consumption of light energy and to induce protective machinery against oxidative stress include non-photochemical quenching (NPQ), photosynthetic control of electron flow between Photosystem II (PSII) and I (PSI), state transitions (ST), cyclic electron flow (CEF), light activation of photosynthetic enzymes in carbon fixation reactions (Calvin–Benson–Bassham cycle, CBB), and induction of antioxidant systems. Recently, the regulatory proteins called thioredoxins (TRX) have been suggested to control many of these mechanisms balancing photosynthetic reactions in chloroplasts.

A covalent and reversible post-translational modification of thiols in side-chains of cysteine residues (Cys) is a common and evolutionarily ancient mechanism to regulate the structure, interactions, or stability and function of proteins. The most common thiol modification is the reversible formation and cleavage of disulfide bonds between redox-active Cys in proteins. The latter reaction is catalyzed by small dithiol:disulfide oxidoreductases called TRXs that are present in all extant lineages of organisms [1]. The classical TRXs contain a redox-active cysteine pair in a highly conserved amino acid motif WCG/PPC [2,3]. The reaction mechanism of TRXs includes a nucleophilic attack on one of the cysteines in a target protein by the catalytic cysteine in the TRX redox-active motif. This attack forms a mixed disulfide between TRX and target protein. Subsequently, the second, resolving cysteine in the TRX reduces the mixed intermolecular disulfide resulting in the release of reduced target protein(s) and oxidized TRX [4,5]. Oxidized TRX is reactivated through reduction by a specific enzyme called thioredoxin reductase (TR). A TRX and a corresponding TR constitute a thioredoxin system.

Two TRX systems exist in plant chloroplasts with different sources of reducing power. Ferredoxin-thioredoxin reductase (FTR) is involved in the ferredoxin-thioredoxin (Fd-TRX) system and activates the classical chloroplast TRXs by receiving reducing equivalents from photosynthetically reduced ferredoxin [6,7]. Plant chloroplasts contain multiple types of TRXs: two f, four m, and two y isoforms as well as x- and z-type of TRX, and several thioredoxin-like proteins (see recent reviews [3,8,9]). In addition to the Fd-TRX system, chloroplast has the NADPH-dependent chloroplast thioredoxin reductase (NTRC) that is reduced by NADPH [10]. NADPH is produced photosynthetically by light reactions but also in the oxidative pentose phosphate pathway (OPPP) that enables the NTRC system to be active also in darkness and under low irradiance [11,12]. Apart from the diverse content of TRXs, the proteomic studies have expanded the range of TRX-regulated plastid processes [13–17]. Besides the early discovery of the activation of the carbon fixation enzymes, TRXs are also now known to control chloroplast biogenesis, plastid transcription, ATP synthesis, photoprotective mechanisms, carbon metabolism beyond the primary photosynthetic reactions, biosynthesis of starch and chlorophyll, nitrogen and sulfur metabolism, the shikimate and OPPP pathways, as well as oxidative stress responses [8,18–20].

In this review, we concentrate on the novel discoveries of TRX-dependent redox-regulation of photosynthetic reactions, both electron transfer and carbon fixation, and metabolism of reactive oxygen species (ROS) strictly related to photosynthesis. We propose that NTRC is an important redox regulator of photosynthesis during the inductive period of dark–light and low–high light transitions and under light intensities that are lower than what plants are acclimated during the growth (e.g. transient shading of leaves in the plant canopy). NTRC is also involved in balancing chloroplast redox poise by being the primary reductant of 2-Cys peroxiredoxins (2-Cys Prx), which scavenge H_2_O_2_ in chloroplasts. Control under the Fd-TRX system prevails under constant illumination of growth and higher light intensities.

## Chloroplast thioredoxin systems

The Fd-TRX system was originally identified and characterized in the 1970s by Bob Buchanan and co-workers (reviewed by ref. [7]). FTR is a heterodimeric enzyme that consists of a catalytic subunit (FTRc), which includes an iron–sulfur cluster [4Fe–4S] and a redox-active motif that mediates electron transfer from Fd to TRXs, as well as a variable subunit (FTRv) [21]. Two isoforms of FTRv exist in Arabidopsis, but their functional significance is unknown [22]. The abundance of all TRXs and FTR is low in comparison with their target proteins [23]. From the TRXs activated by FTR, TRXm1, m2, m4, and TRXf1 are the highest expressed isoforms in leaves [23,24]. TRXx and TRXy2 are expressed at slightly lower levels [24]. The f2, m3, and y1 isoforms as well as TRXz show very low expression in photosynthetic tissues [24].

The Fd-TRX system is essential for plant development and growth, as knockout-mutations of FTRc are lethal, and virus-induced gene silencing (VIGS) of the *FTRB* gene coding for FTRc causes a severe chlorotic phenotype [25] (Table 1). Yet, however, a low FTRc content is enough to maintain a healthy wild-type (WT) phenotype [26]. Oppositely, knockout (KO) mutants of single FTR-dependent TRXs do not have visible phenotypes, and simultaneous mutation of several isoforms is needed to suppress the redundancy of m-type or f-type TRXs [27–30].

**Table 1:** Phenotypes of transgenic lines used to study specificity and cross-talk of the NTRC and Fd-TRX systems in the regulation of photosynthesis

Line (genetic background)	Sp.	Protein(s) affected	Phenotype	References
*ntrc* (WT)	At	NTRC (KO)	Severe impairment of growth and Chl content, high NPQ in low light, impaired reduction in chloroplast enzymes	[10,11,35]
VIGS-*FTRb*	At	FTRc (KD)	Impaired chloroplast development	[25]
*ftrb* (WT)	At	FTRc (KD)	Slight impairment of growth	[26]
*ftra1* or *ftra2* (WT-Ws)	At	FTRv1 (KO) FTRv2 (KO)	Increased sensitivity to oxidative stress	[22]
*trxf1* (WT)	At	TRXf1 (KO)	No visible phenotype	[40]
*trxf1f2* (WT)	At	TRXf1, TRXf2 (KO)	Slight impairment of growth in short day	[29,119]
*trxm1.1* (WT)	At	TRXm1 (KO)	No visible phenotype, decreased activation of NADP+-MDH	[30]
*trxm2.1* (WT)	At	TRXm2 (KO)	No visible phenotype	[30]
*trxm4* (WT)	At	TRXm4 (KO)	Increased NDH-dependent CEF	[46]
*trxz* (WT)	At	TRXz (KO)	Impaired plastid transcription	[58]
*trxm1m2* (WT)	At	TRXm1,m2 (KO)	No visible phenotype, but improved photosynthetic efficiency in low light phases of fluctuating light	[30]
VIGS-*TRXm2m4/m1* (WT or *ntrc*)	At	TRXm1,2, and 4 (KD)	Impaired leaf development, high NPQ More severe phenotype at *ntrc* background	[27,41]
*ntrc trxf1* (WT)	At	NTRC, TRXx (KO)	Very severe impairment of growth and reduction in chloroplast enzymes	[28]
*ntrc trxx* (WT)	At	NTRC, TRXx (KO)	More severe phenotype than in *ntrc*	[42]
*ntrc npq4* (WT)	At	NTRC, PsbS (KO)	Partial recovery of *ntrc* phenotype (lower NPQ)	[69]
*ntrc Δ2cp* (WT)	At	NTRC (KO), 2-Cys Prx A and B (KD)	Partial recovery of *ntrc* phenotype	[79]
OE-NTRC (*ntrc*)	At	NTRC (OE)	Enhanced leaf growth, increased reductive activation of chloroplast enzymes, increased carbon fixation, decreased NPQ, increased CEF and *pmf*	[12,33,34,70]
OE-NTRC (*ndho)*	At	NTRC (OE) NdhO (KO)	No increase in NDH-dependent CEF in the absence of NDH	[12]
OE-NTRC_SAIS_ (*ntrc*)	At	OE of NTRC with inactive NTRd	Partial recovery of *ntrc* phenotype, but exacerbated impairment of reduction in chloroplast enzymes	[33,34]
OE-NTRC_SGPS_ (*ntrc*)	At	OE of NTRC with inactive TRXd	Partial recovery of *ntrc* phenotype, but exacerbated impairment of reduction in chloroplast enzymes	[33,34]
OE-NTRC (WT)	At	NTRC (OE)	Improved stress tolerance	[44]
OE-NTRC (WT)	At	NTRC (OE)	Reduced growth of rosettes	[43]
OE-TRXm (WT)	Nt	TRXfm (OE)	Inhibition of NDH-dependent CEF	[46]

Abbreviations: At, *Arabidopsis thaliana*; Nt, *Nicotiana tabacum*; OE, overexpression; KD, knockdown; KO, knockout.

NTRC forms a complete TRX system in a single polypeptide. NTRC consists of an N-terminal thioredoxin reductase domain (NTRd) with binding sites for NADPH and two flavin adenine dinucleotides (FAD), and of a C-terminal TRX domain (TRXd) [10,11]. The expression of the *NTRC* gene in leaf cells is lower than that of the catalytic subunit of FTR [24]. NTRC functions as a homodimer, where the NTRd of one monomer reduces a disulfide in the TRXd of the other monomer [31–33]. The corresponding site on the TRXd of NTRC facing to NTRd is strongly positively charged, facilitating electrostatic interactions with the oppositely charged surface of the NTRd [33]. Among chloroplast TRXs, the surface charge of the TRXf isoforms most closely resembles the surface charge of TRXd of NTRC [33]. Accordingly, NTRC interacted with TRXf1 in bimolecular fluorescence complementation (BiFC) assays and overexpression of the *NTRC* gene in Arabidopsis enhanced the amount of active TRXf in chloroplasts [34], suggesting that NTRC can donate electrons to TRXf. Additionally, it was recently proposed that NTRC can activate TRXz [26].

The *ntrc* KO line of Arabidopsis has a chlorotic and stunted phenotype, which is particularly severe under short photoperiods [11,35,36]. The *ntrc* chloroplasts are morphologically heterogeneous with size variation and a variable amount of thylakoid membranes [35,37]. It is therefore evident that NTRC has an essential role during early leaf and chloroplast development [38].

## Thioredoxin-dependent regulation of photosynthesis and oxidative stress in chloroplasts

Both *in vitro* and *in vivo* methods including affinity chromatography, fluorescent gel electrophoresis, co-immunoprecipitation, and BiFC have been used to screen for potential proteins targeted to redox regulation [12,13,26,39]. The effect of TRX on the candidate protein activity is then tested by *in vitro* assays with purified TRX and target proteins [26]. These experiments have demonstrated that NTRC and TRXs of the Fd-TRX system are both able to reduce many redox-regulated chloroplast proteins, albeit by different efficiency [26,29,40], suggesting overlapping function of the two chloroplast TRX systems. But, the KO of either chloroplast TRX systems (NTRC, FTR, and several isoforms of TRXm) seriously compromises the photosynthetic activities of mutant plants [10,25,27,30], implying specific functions of the TRX systems that cannot be compensated by the other system. Recently, specificity and functional overlap of the NTRC and Fd-TRX systems have been studied by construction of various combinations of KO mutants lacking NTRC and a distinct type of FTR-dependent TRXs (f-, m-, or x-type TRX) [28,41,42] (Table 1). Transgenic lines overexpressing (OE) a WT or mutated NTRC gene (Table 1) have also disclosed new relationships between the two TRX systems in control of chloroplast redox homeostasis [12,33,34,43,44].

Based on the experimental approaches listed in the previous chapter, both the NTRC and Fd-TRX systems have been proposed to regulate the electron flow in thylakoid membranes (ATP synthase, CEF, NPQ, and ST), enzymes in carbon fixation and starch synthesis, and antioxidant activity in chloroplasts [45–49]. Originally, the Fd-TRX system and TRXf1 were assigned to activate fructose-1,6-bisphosphatase (FBPase), seduheptulose-1,7-bisphosphatase (SBPase), glyceraldehyde-3-phosphate dehydrogenase (GAPDH), and phosphoribulokinase (PRK) in the CBB cycle [6,29,50] as well as the γ-subunit of ATP synthase [51,52]. Recently, the regulation of the CBB cycle also by m-type TRXs has been proposed [53], while initially they were reported to activate the malate dehydrogenase (NADP-MDH) in malate shuttle [54]. TRXx and TRXy2 function in oxidative stress response reactions and in sulfur metabolism [55–57], and TRXz associates with the plastid-encoded RNA polymerase and regulates transcription of plastid genes [58].

NTRC is a primary reductant for 2-Cys Prxs scavenging H_2_O_2_ in chloroplasts [11,34,59–61]. It has also been assigned to activate ADP-glucose-pyrophosphorylase (AGPase) in starch biosynthesis [36,62,63] and several enzymes in chlorophyll biosynthesis [64–66], although a lesser role of NTRC in the regulation of these enzymes has also been proposed [40,67]. Recently, NTRC was reported to regulate the ATP synthase [34,68], NPQ [69,70], and CEF dependent on chloroplast NADH dehydrogenase-like complex (NDH) in thylakoid membranes [12]. Although NTRC has been found to be an inefficient reductant of TRX targets in the CBB cycle in comparison with TRXf and TRXm *in vitro*, deficiency of NTRC *in vivo* impairs the reduction in the enzymes in CBB cycle and photosynthetic electron transfer as well as leaf growth to much greater extent than deficiency of TRXf or TRXm alone [26,28–30].

**Figure 1.**
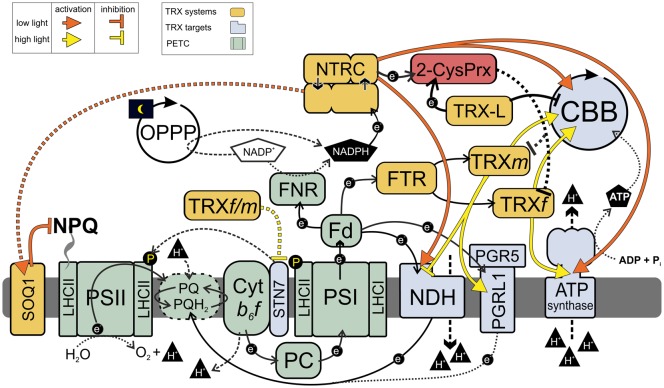
Dynamic regulation of photosynthesis by chloroplast thioredoxin systems. At dark–light and low–high light transitions as well as under light intensities limiting photosynthesis, NTRC activates NDH complex, ATP synthase, and CBB cycle, which helps to balance the redox poise between light and carbon fixation reactions. NTRC may also mediate the SOQ1-dependent down-regulation of NPQ to prevent heat dissipation under low light. Fd-TRX system keeps the redox-regulated photosynthetic enzymes active under moderate and higher light intensities. In relation to NTRC, TRXm has an antagonist role in the regulation of the electron flow in the thylakoid membrane. 2-CysPRXs are involved in the oxidation of chloroplast TRX systems. For details, see the text. Dotted arrows represent hypothetical or potentially indirect effect on photosynthetic proteins. PETC, photosynthetic electron transfer chain.

NTRC and TRXm may also have antagonistic roles in the regulation of chloroplast electron transfer activities (Figure 1). Plants deficient in m-type TRXs show enhanced PSI yield in low light intensities [30]. This is opposite to *ntr*c KO plants but similar to NTRC-overexpressing plants, whose PSI yield is enhanced in low and fluctuating light [12,34]. Moreover, TRXm4 has been proposed to have an inhibitory effect on NDH-dependent CEF [46], while plants overexpressing NTRC enhance NDH-dependent CEF [12]. Furthermore, NTRC overexpression results in dark phosphorylation of light harvesting complex II (LHCII) proteins by the STN7 kinase and, consequently, to an increase in the relative size of the PSI antenna cross-section in dark-adapted leaves, as well as enhanced efficiency of ST [70]. In contrast, overexpression of TRXm in tobacco inhibited STN7-dependent phosphorylation of LHCII proteins and ST, while overexpression of TRXf had no effect on these processes [71]. The specific targets (single or multiple proteins) of this antagonist regulation by NTRC and TRXm remain to be elucidated in the further studies.

## NTRC and Fd-TRX systems are differentially activated by light conditions

Instead of strictly distinct tasks with separate target proteins, the two TRX systems may build up a complex redox network in chloroplast with partial specificity but also partially overlapping functions, which facilitates plant growth under repetitively changing environmental conditions.

The Fd-TRX system is tightly coupled to photosynthetic electron transfer activity, and thus, it has been regarded as a primary activator of photosynthetic reactions in light. However, while the Fd-TRX system is activated rapidly under high irradiance [72], it is mostly oxidized in darkness and under low light [29,34,72]. Therefore, initial activation of photosynthetic processes at dark–light transitions and adjustment of plastid redox homeostasis in low light conditions and during sudden changes in irradiance requires a redox regulator capable of being active under these conditions. NTRC is an ideal candidate because it is activated by NADPH present both in dark-adapted and illuminated chloroplasts. Accordingly, it has been demonstrated that the NTRC pool is partially reduced in darkness and the amount of reduced NTRC also stays fairly constant irrespective of light intensity, as well as during dark–light transitions [12]. This unique property of NTRC allows it to function as an effective redox regulator in conditions where light or developmental stage of the chloroplast limits the activation of the Fd-TRX system by the photosynthetic electron transfer chain (PETC) (Figure 1).

How can NTRC act as a dynamic regulator of photosynthetic processes in response to changes in environmental conditions if its own redox state is fairly constant? There are different options to explain this discrepancy. The affinity of the TRX to target proteins may change considerably in different physiological conditions of chloroplasts, such as stromal pH and ion concentrations, which may affect the activation states and target specificities of TRXs [73,74]. For example, NTRC, whose midpoint redox potential has been measured *in vitro* as −275 mV [26], is the primary reductant of 2-Cys Prxs [11,60], whose redox potential *in vitro* is as low as −315 mV [75]. Thus, the reduced NTRC form may only be able to reduce some of its targets in specific circumstances.

Alternatively, the access of NTRC to its substrates may be partly controlled by the redox state of the Fd-TRX system. The f- and m-type TRXs have more negative redox potentials and a higher affinity to common TRX targets *in vitro* than NTRC [26,76,77]. The combined amount f- and m-type TRXs in the chloroplast is about six times higher than that of NTRC [78]. When active, the Fd-TRX system probably competes with NTRC for interaction with common targets. NTRC-mediated reduction would therefore be required when the activation state of the Fd-TRX system is low, such as in darkness and low light conditions [12,34]. This hypothesis would also explain the discrepancies between *in vitro* and *in vivo* experiments of TRX target specificity. NTRC has been found to be an inefficient reductant of TRX targets in the CBB cycle in comparison with TRXf and TRXm *in vitro*, but *in vivo*, deficiency of NTRC impairs the reduction in the enzymes in CBB cycle, photosynthetic electron transfer and leaf growth to much greater extent than deficiency of TRXf or TRXm [26,28–30,34]. These studies demonstrate that NTRC has a specific function in the regulatory network of the chloroplast that cannot be compensated by other TRXs.

## Regulation of photosynthetic processes by NTRC allows the maintenance of redox homeostasis at dark–light transitions and during changes in light conditions

We propose that the NTRC system operates as a vital regulatory hub that couples the metabolic state of the stroma to the redox poise of the PETC in low light conditions and during fluctuations in light intensity (Figure 1). This is achieved by simultaneous redox control of the induction of NPQ and the activity of the ATP synthase, cyclic electron transfer around PSI through the NDH complex, and CBB cycle enzymes [12,30,34,68–70]. Under growth and higher light intensities, the Fd-TRX system is fully activated and can take over the redox-regulation of photosynthesis.

Reduction in γ-subunit of the ATP synthase by the f-type TRX of the Fd-TRX system is well established [48,52]. However, the Fd-TRX system can only compensate for a lack of NTRC in moderate to high light conditions, while in low light NTRC deficiency results in impaired reduction in the γ-subunit, with several consequences [34,68]. Firstly, activity of the ATP synthase remains low in *ntrc* plants resulting in the acidification of the lumen that, in turn, induces high NPQ [12,34,69]. Moreover, low activity of the ATP synthase results in low ATP production, which together with impaired reduction in CBB cycle enzymes [26,34,79] contributes to a decreased rate of carbon fixation in low light in NTRC-deficient plants [11,34,35,60].

The rate of CEF also depends on the stromal redox state [49,80]. There are two CEF pathways in plant chloroplasts, one dependent on the proteins proton gradient regulation 5 (PGR5) and PGR5-like 1 (PGRL1) [81,82], and the other dependent on the chloroplast NDH complex [83,84]. PGRL1 has been proposed to form a heterodimer with PGR5 that oxidizes ferredoxin and reduces the plastoquinone (PQ) pool [47]. During dark–light transitions, PGR5/PGRL1-dependent CEF contributes to generation of proton motive force (pmf) that induces photoprotective mechanisms, NPQ, and photosynthetic control [47,81,82,85–87]. PGRL1 contains six conserved Cys residues, which have been proposed to control the conformation of PGRL1 protein and interaction with PGR5 [47,81,82,88]. According to a model postulated by Dario Leister and co-workers, TRXm activates the reduction in the PQ pool by PGR5/PGRL1 proteins [47,89]. PGRL1 protein has, indeed, been shown to be transiently reduced during dark–light transitions, coinciding with an increase in P700 oxidation and NPQ induction [12,47]. Alternatively, it has been suggested that PGR5 alone may down-regulate the proton conductivity of the thylakoid membrane upon sudden increases in light intensity via inhibition of ATP synthase activity [90,91]. This down-regulation is enhanced by NTRC overexpression, but not in the absence of PGR5 [12]. As NTRC was shown to interact with PGR5 [12], it is likely that the function of PGR5 and PGRL1 depends on the stromal thiol redox state.

It has been suggested that NDH-mediated CEF is involved in balancing photosynthetic redox poise and generation of pmf specifically in low light conditions and during increases in light intensity [92–95]. The NDH complex is reduced by Fd [96], which functions as a light-dependent redox hub, controlling the distribution of electrons to several stromal acceptors in addition to CEF pathways [97]. Therefore, strict regulation of NDH activity is likely needed for concerted function of all these processes under fluctuating light conditions, in order to maintain redox balance in the chloroplast. It has been shown by KO and overexpression lines of NTRC that it has a role in activating the NDH complex during dark–light transitions and under low irradiance [12]. NTRC overexpression enhances reduction in the PQ pool in darkness, increases the magnitude of pmf and PSI yield in comparison with WT in low light and upon increases in light intensity, and enhances the acidification of the lumen under all light intensities in an NDH-dependent manner [12]. These observations suggest that in WT plants, NTRC-dependent activation of NDH at dark–light and under fluctuating light releases redox pressure in thylakoid membranes by inducing dissipation of light energy as heat by NPQ until the CBB cycle is ready to exploit the electrons in carbon fixation.

The major, energy-dependent component (qE) of NPQ depends on acidification of the lumen, via protonation of the PSII subunit S (PsbS) protein and association of the xanthophyll cycle enzyme violaxanthin de-epoxidase with the thylakoid membrane [98–100]. As reviewed in the previous chapters, both deficiency and increase in NTRC content *in vivo* cause higher acidification of thylakoid lumen in illuminated leaves, albeit for different reasons (see discussion in ref. [12]). High lumen acidification already at low light intensities correlates with the exceptionally high NPQ reported in *ntrc* plants [68–70]. However, *ntrc* has high NPQ also under growth light and higher intensities, despite the level of ΔpH being comparable to WT in those conditions [70]. But, generation of NPQ is diminished in leaves overexpressing NTRC despite strong acidification of the lumen and higher accumulation of xanthophyll pigments [70]. These observations indicate that an unknown factor independent of trans-thylakoid ΔpH is up-regulating NPQ in *ntrc* and down-regulating it in plants overexpressing NTRC. Inhibition of a slow-relaxing component of NPQ has been shown to depend on SUPPRESSOR OF QUENCHING 1 (SOQ1), an integral thylakoid membrane protein that contains a lumenal thioredoxin-like domain [101,102]. Importantly, this component called qH [102] is ΔpH-independent, as in addition to *soq1* KO plants, a slow-relaxing NPQ component remains elevated in plants lacking both SOQ1 and PsbS [101]. A similar slowly reversible NPQ component was detected also in *ntrc* [70], and SOQ1 was identified as a putative NTRC interactor by co-immunoprecipitation/MS [12]. Thus, the absence of NTRC may impair SOQ1-dependent down-regulation of NPQ, while NTRC overexpression might result in over-activation of this inhibitory mechanism. It has been reported that the mutation of PsbS in the *ntrc* background partially restored the photosynthetic activity of the double mutant by reducing NPQ [69], indicating that the *ntrc* mutant suffers from energy shortage because of the uncontrolled NPQ. Thereby, the task of TRX may be to moderate the induction of NPQ under light intensities limiting photosynthesis.

Impaired and enhanced carbon fixation rates in *ntrc* and plants overexpressing NTRC, respectively [11,34,35], suggested that the activation states of the redox-regulated enzymes in the CBB cycle are either directly or indirectly affected by deficiency and overexpression of NTRC. Indeed, reduction in FBPase and PRK was impaired in low light conditions in *ntrc* [34,79], while the *in vivo* amount of reduced forms of these enzymes was significantly higher than in WT in all light conditions and even in dark-adapted leaves of OE-NTRC plants [34]. As both NTRC and TRXf1 interact with FBPase and PRK in BiFC [34], it is likely that at least these CBB enzymes are directly and cooperatively regulated by both TRX systems.

In summary, the NTRC- and FTR-mediated regulation of photosynthetic redox poise is presented in Figure 1. Upon the onset of illumination of leaves, the NTRC pool is already partially active due to NADPH produced in the OPPP in darkness. It transiently activates CEF that helps to balance redox poise in thylakoid membranes before activation of the CBB cycle. Under light intensities limiting photosynthesis, NTRC activates the ATP synthase by reducing the γ-subunit and induces ATP production. NTRC also contributes to activation of the CBB cycle, which together with activation of NDH-dependent CEF enhances the electron sink capacity of the stroma and alleviates acceptor side limitation of PSI. Under low irradiance, NTRC may also mediate SOQ1-dependent down-regulation of NPQ [70,102]. Under growth light and higher irradiance, the m-type TRXs are involved in the regulation of PGRL1/PGR5-dependent CEF [12,47]. All the events described here contribute to the prevention of over-reduction in the electron transfer chain and allow efficient oxidation of PSI, protecting it from photodamage [103].

## Oxidation of chloroplast TRX and redox-regulated proteins

Redox regulation of chloroplast proteins is reversible, including also the oxidation loop of TRX systems and target proteins. Much less, however, is known about the mechanisms oxidizing TRXs and proteins, especially under fluctuating light or at light–dark transitions. Molecular oxygen and ROS have been reported to oxidize protein thiols [6,104], and they are probably involved in adjustment of the redox state of proteins regulated by TRXs. Since the oxygenic photosynthetic organisms evolve oxygen and produce ROS in light, continuous supply of reducing equivalents from TRX systems is needed to keep the photosynthetic enzymes active in light. Accordingly, molecular oxygen and ROS can be involved in oxidation of TRXs and enzyme pools also when light intensity drops.

Recently, involvement of 2-Cys Prxs in oxidation of TRX systems and redox-regulated proteins has been reported (Figure 1) [43,79,105–107]. 2-Cys PrxA and B are highly abundant chloroplast proteins that scavenge peroxides in chloroplasts [23,78]. NTRC is a primary reductant for 2-Cys Prxs, but they can also be reduced by other chloroplast TRXs, including TRXf, TRXx, and TRX-like proteins ACHT1 and ACHT4 [11,77,108]. Danon and his colleagues [105,108] have described an oxidizing loop of TRX system regulating AGPase, a key enzyme in starch synthesis under fluctuating light. ACHT4 is first reduced in light but becomes then oxidized by 2-Cys Prxs. Oxidized ACHT4 inactivates AGPase at light/dark transition or under fluctuating light. Chloroplast TRXs have also been suggested to become oxidized by an oxidized pool of 2-Cys Prxs [79,106]. As highly abundant proteins, 2-Cys Prxs can form a very strong sink for electrons when ROS accumulate in chloroplasts. This strong sink abstracts electrons from TRX systems involved in the activation of redox-regulated chloroplast enzymes [79,106]. Accordingly, it was proposed that the impaired reduction in CBB cycle enzymes in *ntrc* results indirectly from the oxidation of TRXf in light due to high accumulation of oxidized 2-Cys Prxs [79]. An atypical TRX called Thioredoxin-like2 was also suggested to mediate the oxidative effect of 2-Cys Prxs on chloroplast enzymes [107]. However, contrary to [79], reduction in TRXf was not impaired in *ntrc*, or in transgenic lines overexpressing mutated NTRC lacking TRX activity, despite these lines have substantially larger pools of oxidized 2-Cys Prxs in comparison with WT in all tested light conditions [34]. But, higher amounts of active CBB cycle enzymes accumulate in illuminated leaves in mutants with strongly diminished amounts of 2-Cys Prx proteins [79], as well as in illuminated plants overexpressing NTRC, which accumulate low amounts of oxidized 2-Cys Prxs [34]. Both NTRC overexpression [12] and 2-Cys Prx deficiency [106] result in enhanced photosynthetic efficiency in fluctuating light conditions. Thus, 2-Cys Prxs are probably involved in an oxidizing loop of TRX systems in the chloroplast by controlling the amount of redox equivalents available for redox-regulated photosynthetic enzymes (Figure 1). How efficient and dynamic the 2-Cys Prxs-dependent oxidant system is in the control of photosynthetic enzymes remains to be elucidated.

## Localization of photosynthetic and regulatory proteins in mesophyll and bundle sheath cells of C4 leaves

Chloroplast TRX systems described above are applied to C_3_ plants primarily fixing CO_2_ via the CBB cycle. In C_4_ plants, two different chloroplast populations exist with the segregation of phosphoenolpyruvate carboxylase (PEPC)-dependent carbon fixation into mesophyll cells (MCs) and ribulose-1,5-bisphosphate carboxylase/oxygenase (Rubisco)-dependent carbon fixation into bundle sheath cells (BSCs). Three biochemical subtypes of C_4_ photosynthesis have been described with variations of C_4_ photosynthetic enzymes, transporters, and metabolites transported between MCs and BSCs (see the recent review by Furbank [109]). The proteomic approach has demonstrated remarkable differences in the composition of photosynthetic proteins in MS and BSC chloroplasts of maize, which belongs to the C_4_ subtype called NADP malic enzyme (NADP-ME) [110,111]. Maize MC chloroplasts have less CBB cycle enzymes except for the enrichment of GAPDH and triose phosphate isomerase (TPI) catalyzing the reduction phase of 3-phosphoglycerate to triose phosphates, whereas the other CBB cycle enzymes are enriched in BSCs [111]. Subunits of PSII and LHCII are enriched in MCs, while BSC chloroplasts lack the grana structure and accumulate slightly more subunits of PSI, LHCI, cytochrome bf_6_ complex (Cytbf_6_), and ATP synthase than MC chloroplasts [110]. Consistent with the divergent localization of PSI and PSII complexes between MCs and BSCs, the components regulating linear electron transfer (NPQ, ST) and mediating CEF (NDH) are enriched in MCs and BSCs, respectively [110,112]. Not only are the subunits of the NDH complex enriched in BSCs but also the total abundance of NDH is higher in C_4_ than in C_3_ leaves, suggesting that NDH supports the high CEF activity observed in C_4_ leaves [112]. The components of the other CEF pathway involving PRG5/PGRL1 proteins show a different pattern. In maize leaves, PGRL1 is enriched in BSCs, while PGR5 is equally localized to both cells [111], while both proteins are equally present in MCs and BSCs of C4 *Flaveria* species, albeit PGRL1 and PGR5 proteins are more abundant in C_4_ than in C_3_ *Flaveria* species [112].

Although chloroplast TRX systems are important activators of the CBB cycle in C_3_ species, the components of the both chloroplast TRX systems and other TRX-like proteins and glutaredoxins are enriched in maize MCs having only traces of CBB cycle enzymes [111]. Especially both TRs, FTR and NTRC are largely localized to MCs (protein ratio in BSC/MC 0.14 and 0.09, respectively). The m-type TRXs and 2-Cys Prxs are, however, more equally distributed in MCs and BSCs. Only CDSP32, a TRX-like drought-induced stress protein [113] is highly enriched in maize BSC chloroplasts [111]. The low content of FTR-dependent TRXs in maize BSCs is probably related to low O_2_ concentration caused by strong deficiency of PSII and low ROS production due to the almost complete lack of linear electron flow. Accordingly, also the antioxidant proteins are mainly enriched in maize MCs, except for stromal ascorbate peroxidase, which is more abundant in BSCs [111]. This segregation of TRX, antioxidants, linear and CEF, and C_3_/C_4_ carbon fixation between MCs and BSCs suggests that the demand of TRXs is strongly linked with the presence of molecular oxygen and ROS. Due to the higher oxidizing capacity, continuous relay of electrons from TRX systems to redox-regulated photosynthetic proteins is needed to maintain the active state of the enzymes in illuminated maize MC chloroplasts producing molecular oxygen and ROS. Interestingly, the enzymes catalyzing the electron sinks for linear electron flow in MCs, i.e. reductive phase of CBB cycle (GAPDH and TPI) and the reduction in oxaloacetate to malate in C4 shuttle (NADP-MDH), are both enriched in MCs and potentially (TPI [114], GAPDH [13]), or confirmed (NADP-MDH [115]) to be targets of TRX regulation. In the previous chapters, we proposed that TRXs in C_3_ chloroplasts are involved in balancing electron transfer activity in thylakoid membranes with the electron sinks in stroma under fluctuating light conditions. Accordingly, MC chloroplasts probably need to control the redox homeostasis between source and sink of electrons, as observed in C_3_ chloroplasts.

How are the redox-regulated enzymes of the CBB cycle activated in BSCs, when both the NTRC and Fd-TRX systems are present as very low concentrations? BSC chloroplasts contain m-type TRXs, and probably this is enough to keep the enzymes active in light, when the production of oxidants that inactivate redox-regulated enzymes is low. Furthermore, the high CO_2_ concentration in BSCs makes a strong sink for ATP and NADPH, further lowering the probability of oxidant production in light reactions. The role of CDSP32 and ascorbate peroxidase in BSC remains to be elucidated by further studies.

## Can we improve plant photosynthesis by overexpressing NTRC?

Chloroplast TRX systems are engaged in the complex redox network that balances photosynthetic redox poise and consumption of electrons under fluctuating light. This regulatory network helps sessile plants to survive in nature under ever-changing environmental conditions. The novel information about the structure, function, and regulation of photosynthesis has now revealed potential routes to increase photosynthetic efficiency and thus productivity of photosynthetic organisms by manipulating the regulatory network in chloroplasts [116,117]. Crop plants form tight canopies with sun-exposed and shaded leaves that repeatedly face fluctuation of light. The upper canopy exposed to the sun has reported to drive ∼75% of photosynthesis [118], while the photosynthetic efficiency of the rest of the leaves depends largely on sun flecks. The shaded leaves cannot efficiently utilize sunflecks because the increase in light intensity rapidly induces the mechanisms dissipating light energy as heat due to the delay in the activation of CBB cycle enzymes [117]. Thus, the mechanisms that down-regulate photosynthetic activities at light intensity transitions and at low light are potential targets for manipulation in order to improve photosynthetic efficiency of plants.

The effect of an increase (overexpression) or reduction (KO/knockdown) of a single component of the regulatory mechanism depends on how significant the mechanism is for photosynthetic performance and what are the side effects of the modification. So, the regulator that forms a node in the regulatory network and mediates signals to various processes in photosynthesis may be a promising candidate for manipulation, if it has a parallel and positive effect on photosynthetic subreactions. We have proposed that NTRC may fulfill these criteria as a general positive effector of photosynthetic activities. The NTRC system broadly activates photosynthetic reactions including ATP synthesis, CEF, and carbon fixation at low and fluctuating light, as described in the previous chapters. It is also involved in scavenging of H_2_O_2_ produced in light reactions [11] and in down-regulation of NPQ under conditions where the dissipation of energy as heat would be wasteful [69].

Contradictory reports have been published about the effect of NTRC overexpression on growth of Arabidopsis (Table 1). Arabidopsis lines overexpressing NTRC were constructed by transformation of the *ntrc* mutant with the WT *NTRC* gene under a constitutive 35S-CaMV promoter, and only the transgenic plants with fully-complemented WT phenotype were selected for further studies. The NTRC protein content of these lines was ∼10–20 times higher than in WT Arabidopsis [12,33,34]. In these lines, overexpression of NTRC significantly increases biomass production of Arabidopsis rosettes [33]. Accordingly, Kim et al. [44] have reported on higher tolerance of Arabidopsis plants overexpressing NTRC to oxidative and drought stresses. Neither growth defect was observed in these OE-NTRC lines. However, Ojeda et al. [43] reported reduced growth in two OE-NTRC lines, which were constructed by transformation of WT Arabidopsis with the *NTRC* gene under a 35S-CaMV promoter. The increase in the amount of NTRC was not reported in this paper, but the immunoblot shows substantially higher accumulation of NTRC in these transgenic lines in comparison with previously constructed lines, which may explain the different growth effect of the OE-NTRC lines. The large increase in NTRC content may considerably disturb redox homeostasis in chloroplasts, e.g. by imbalancing the NADP^+^/NADPH ratio. Secondly, 35S-CaMV promoter causes an ectopic expression of the gene also in non-photosynthetic tissues that endogenously have a low amount of NTRC [24], which may seriously impede the cellular function of these tissues.

In the transgenic lines containing a moderately increased amount of NTRC, photosynthetic carbon fixation was raised ∼20% [34]. They have also higher activity of CEF [12] and permanently elevated activation states of the CBB cycle enzymes (FBPase and PRK in ref. [34]) (GAPDH in ref. [43]), which creates a strong sink for electrons from PSI. This allows faster balancing of photosynthetic redox poise and efficient electron transfer upon onset of illumination or under fluctuating light. Hence, the quantum yield of PSI and CO_2_ fixation was higher than in WT immediately upon illumination in dark-adapted OE-NTRC leaves and under low light [12,34]. Changes in the NTRC content of leaves also affected a wide variety of chloroplast functions indirectly through cross-talk with the Fd-TRX system and by generally modifying the stromal thiol-redox state [34,43]. The higher accumulation of active TRXs of the Fd-TRX system in plants overexpressing NTRC may be due to the direct reduction in these TRXs by NTRC or to a very small pool of oxidized 2-Cys Prxs in the leaves with elevated NTRC content under all tested light intensities [34]. Better stress tolerance of OE-NTRC plants [44] would also improve the fitness of plants under natural growth conditions. As the redox state of the NTRC pool in leaves moderately overexpressing NTRC was maintained fairly constant in all light conditions [12], these results suggest that overexpression of NTRC is a simple genetic modification that could considerably improve photosynthetic efficiency and biomass yield in shaded leaves and in fluctuating light conditions. To avoid harmful side effects due to the too high NTRC content or ectopic expression of *NTRC* gene in non-photosynthetic tissues, the overexpression construct with leaf- and light-specific promoters should be tested. The potential of TRX overexpression as a bioengineering tool to improve the yields of crop plants or biofuel production seems, however, to be worth considering.

## Abbreviations

2-Cys Prx: 2-Cysteine peroxiredoxin
AGPase: ADP-glucose-pyrophosphorylase
At: *Arabidopsis thaliana*
BiFC: bimolecular fluorescence complementation
BSC: bundle sheath cell
CBB cycle: Calvin–Benson–Bassham cycle
CEF: cyclic electron flow
Chl: chlorophyll
Cys: cysteine residue
Cyt b_6_f: cytochrome b_6_f complex
FAD: flavin adenine dinucleotide
FBPase: fructose-1,6-bisphosphatase
Fd: ferredoxin
Fd-TRX system: ferredoxin-dependent thioredoxin system
FNR: ferredoxin-NADP^+^ oxidoreductase
FTR: ferredoxin-thioredoxin reductase
FTRc: catalytic subunit of FTR
FTR_v_: variable subunit of FTR
GAPDH: glyceraldehyde-3-phosphate dehydrogenase
KD: knockdown
KO: knockout
LHCII: light harvesting complex of PSII
MC: mesophyll cell
NADP-MDH: malate dehydrogenase
NDH: chloroplast NADH dehydrogenase-like complex
NPQ: non-photochemical quenching
Nt: *Nicotiana tabacum*
NTRC: chloroplast NADPH-dependent thioredoxin reductase
NTRd: N-terminal thioredoxin reductase domain
OE: overexpression
OPPP: oxidative pentose phosphate pathway
PETC: photosynthetic electron transfer chain
PGR5: proton gradient regulation 5
PGRL1: PGR5-like 1
pmf: proton motive force
PQ: plastoquinone
PRK: phosphoribulokinase
PsbS: PSII subunit S
PSI: photosystem I
PSII: photosystem II
qE: energy-dependent component of non-photochemical quenching
ROS: reactive oxygen species
SBPase: seduheptulose-1,7-bisphosphatase
SOQ1: suppressor of quenching 1
ST: state transitions
TPI: triose phosphate isomerase
TR: thioredoxin reductase
TRX: thioredoxin
VIGS: virus-induced gene silencing
WT: wild type
